# Empowering Executive Functions in 5- and 6-Year-Old Typically Developing Children Through Educational Robotics: An RCT Study

**DOI:** 10.3389/fpsyg.2019.03084

**Published:** 2020-02-05

**Authors:** Maria Chiara Di Lieto, Chiara Pecini, Emanuela Castro, Emanuela Inguaggiato, Francesca Cecchi, Paolo Dario, Giovanni Cioni, Giuseppina Sgandurra

**Affiliations:** ^1^Department of Developmental Neuroscience, IRCCS Fondazione Stella Maris, Pisa, Italy; ^2^Department of Education, Languages, Intercultures, Literatures and Psychology, University of Florence, Florence, Italy; ^3^Institute of BioRobotics, Sant’Anna School of Advanced Studies, Pisa, Italy; ^4^Department of Clinical and Experimental Medicine, University of Pisa, Pisa, Italy

**Keywords:** educational robotics, executive functions, response inhibition, working memory, children

## Abstract

Educational Robotics (ER) is a new learning approach that is known mainly for its effects on scientific academic subjects such as science, technology, engineering, and mathematics. Recent studies indicate that ER can also affect cognitive development by improving critical reasoning and planning skills. This study aimed to quantify the ability of ER to empower Executive Functions (EF), including the ability to control, update, and program information, in 5- and 6-year-old children attending first grade, a crucial evolutionary window for the development of such abilities. A total of 187 typically developing children were enrolled and randomly allocated into two experimental conditions: A, for immediate ER training, and B, for waitlist. ER-Laboratories (ER-Lab) for small groups were organized at schools, using a child-friendly, bee-shaped robot called Bee-Bot^®^ (Campus Store). Activities were intensive, enjoyable, and progressively more challenging over the 20 twice-weekly sessions. Outcome measures, based on standardized tests, were used to quantify the effects of ER on EF. Compared to the control group, the ER-Lab group showed significantly better ability to actively manipulate information in short-term memory and suppress automatic responses in favor of goal-appropriate actions. This RCT study provides the first quantitative evidence of the positive effects of ER activities for improving working memory and inhibition in the early school years.

## Introduction

Educational Robotics (ER) refers to a learning approach requiring students to design, assemble, and program robots through play and hands-on activities. ER was developed in the 60 s through the integration of psycho-pedagogical cognitive development theories (Piaget and Inhelder, 1966; Papert, 1980) and social learning theories (Vygotsky, 1978; Bandura, 1986). ER creates a learning environment where students can simultaneously interact with peers and robots. Most ER studies conducted in schools have focused on examining the impact of ER activities on the “STEM” areas (Science, Technology, Engineering, and Mathematics), with particular focus on robot design and assembly (Hussain et al., 2006; Barker and Ansorge, 2007; Nugent et al., 2008, 2010). Other studies have examined using ER as an assistive device for improving motor and social-communication problems (Krebs et al., 2012; Vanderborght et al., 2012; Srinivasan et al., 2016). Recent studies have assessed the effects of robot programming on cognitive and learning processes, such as auto-monitoring, attention, decision-making, problem-solving, and computational thinking (Highfield, 2010; La Paglia et al., 2011; Kazakoff and Bers, 2014). Nevertheless, most of the studies lacked experimental designs or quantitative outcome measures; thus, it is still unclear which cognitive functions may be significantly improved through ER during childhood (Benitti, 2012; Alimisis, 2013).

Recently, we conducted a pilot study to measure how ER can improve cognitive and learning abilities in preschool children (Di Lieto et al., 2017). An intensive laboratory [ER-Laboratories (ER-Lab)] was conducted for 6 weeks using a bee-shaped robot, called Bee-Bot^®^, incrementally introducing more difficult robot programming activities. The children were assessed with standardized tests, and the results showed that ER-Lab activities promoted some superior cognitive functions, such as Executive Functions (EFs). Robot programming requires children to mentally plan a complex sequence of actions before the motor act: first the child had to set the target or targets to reach, then to plan the sequential steps needed to arrive at the target, and finally, at the end of the programming, to act and verify his or her behavior. Several complex superior cognitive functions are involved in this type of task, such as abstraction and logical reasoning, decision-making, sequential thinking, maintaining and updating information in memory, and problem-solving, all functions that concern the EFs cognitive domain. There is agreement in the literature that EFs represent a group of top-down processes that are important for adaptive and goal-directed behavior (Miyake et al., 2000; Lehto et al., 2003). However, several controversies exist regarding defining and differentiating separable EF components during the course of development because we now recognize the internal complexity of each factor and the unity and diversity of the different EF components (Miyake et al., 2000; Howard et al., 2014; Friedman and Miyake, 2017; Karr et al., 2018; Morra et al., 2018). Within a developmental perspective, the model proposed by Diamond (2013) is largely used. This model consists of three main EF factors: inhibition, working memory, and cognitive flexibility, which are strongly related to more complex EFs, such as reasoning, planning, and problem-solving. Following Diamond’s definitions, inhibition represents a complex construct theorized as a set of functions rather than as a unitary construct, distinguishing response inhibition at the behavior level from interference control at the memory, thoughts, and attention levels; working memory involves holding visual or verbal information in mind and mentally working with it; and cognitive flexibility is the ability to efficiently change spatial and interpersonal perspectives.

Executive Functions develops over time and are completed during late adolescence (Garon et al., 2008). Pre-school and primary school are critical times for EFs maturation and are linked to attaining academic milestones (Diamond, 2013). EF development consists of both quantitative and qualitative changes. Some studies suggest that, in toddlers, there is an undifferentiated executive control factor, while a two-factor model consisting of inhibition and working memory emerges between 3 and 5 years (Miller et al., 2012). Another two-factor model where inhibition is distinguished from working memory and shifting (which partially resembles the cognitive flexibility component of Diamond’s model) has been identified in 5- and 6-year-old children, followed by the emergence of a separate three-factor structure later in development (Usai et al., 2014). However, these trajectories are not universally supported, and results from a recent systematic review (Karr et al., 2018) show that no model consistently converges across samples but that there is evidence for greater EFs unidimensionality among child/adolescent samples. Disentangling the various hypotheses on the developmental EFs structure is beyond the purpose of the present study, but the types of tasks and tests used in the different studies may have contributed to the high variability of the results (Miller et al., 2012). Both the EF models and the measures used could affect the methodological choices and results obtained in intervention studies on enhancing EF development (Diamond and Lee, 2011).

Most previous studies that are focused on improving EFs during development differ from those focused on clarifying EFs structure and ways of measuring the different EF components; nevertheless, some general principles useful for intervention studies have been developed. In particular, recent studies suggest that EFs can be trained, and, to obtain significant changes, the training needs to: (1) create incrementally more challenging activities based on adaptive and intensive paradigms, as demonstrated by studies on home-based software (Thorell et al., 2009), (2) be administered over long training phases, especially for very young participants, (3) continuously monitor participation levels (Wass, 2015; Diamond and Ling, 2016), (4) constantly challenge EFs to produce improvements (Diamond and Ling, 2016), (5) provide different and heterogeneous training tasks serving the same purpose (Rueda et al., 2005; Wass et al., 2011) or targeting similar cognitive mechanisms (Klingberg et al., 2005), and (6) plan enjoyable and social activities because benefits will be greater if emotional, social, and physical needs are also addressed (Diamond and Ling, 2016).

According to the principles listed above, this study, which is part of a wider research project called “e-Rob,” aimed to enhance EFs in first-grade children through in-school ER-Lab by means of enjoyable, intensive, and incrementally more challenging activities requiring students to program a bee-shaped robot called Bee-Bot^®^ (Campus Store). Based on a previous pilot study on a small sample of preschoolers, the present research aimed to bring further evidence to the hypothesis that ER-Lab may induce positive effects in visuospatial working memory and inhibition during a critical period of development.

## Materials and Methods

### Participants

A total of 187 typically developing first-graders (90 females, 97 males; age range from 5 years and 6 months to 6 years and 8 months) were selected to participate in ER-Lab. Enrollment was conducted in collaboration with the District of Pisa to contact as many schools as possible. Thirteen classes from nine schools were enrolled, from which 187 children with typical development and 42 children with special needs were selected. To comply with the aims of this study, only data collected from typically developing children are reported and discussed (see Table 1 for details on the number of children and teachers involved in each class).

**TABLE 1:** Number of children and teachers involved in each school and class.

School	Class	Number of enrolled teachers	Number of enrolled typically developing children
School 1	Class 1	2	12
	Class 2	2	11
School 2	Class 3	2	23
School 3	Class 4	4	15
School 4	Class 5	2	15
	Class 6	2	11
	Class 7	2	11
School 5	Class 8	2	16
School 6	Class 9	2	17
	Class 10	2	18
School 7	Class 11	2	7
School 8	Class 12	2	20
School 9	Class 13	2	11
TOTAL		28	187

This research project was approved by the Pediatric Ethics Committee of the Tuscany Region. All parents gave written consent for their child’s participation and for publication of the results.

### ER-Lab Training

**FIGURE 1:**
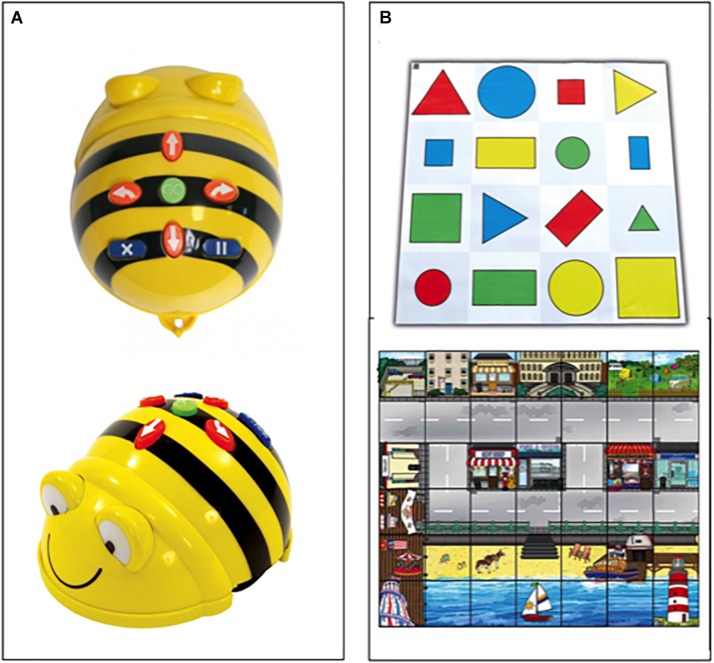
**(A)** The Bee-Bot and **(B)** some examples of colorful carpets.

ER-Lab was conducted twice a week for 10 weeks (20 ER training sessions of 60 min) using the Bee-Bot robot (Bee-Bot^®^, Campus Store). The design of Bee-Bot is child-friendly, with a black/yellow bee shape, sounds, and lights that make it very attractive for children (Figure 1A). The Bee-Bot can be programmed with up to 40 instructions in a single program using buttons on its back to program motion or rotation. Four orange buttons move the robot either forward or backward (15 cm) and rotate it right or left (90° rotation); a central green button (GO button) starts the programmed sequence; a blue button clears the memory (CLEAR or X); and another blue button programs a short pause during robot motion (PAUSE or II). The user cannot modify the length of steps or degree of angular rotation. At the end of the programmed sequence, Bee-Bot provides visual and acoustic feedback. To guide robot programming and sustain motivation, different colorful carpets, characterized by a 15 × 15 cm matrix, were provided (Figure 1B).

Small groups of five or six children were formed for each ER-Lab; each group had two Bee-Bots and a carpet. Two teachers and one experimenter in each class guided and participated in the ER-Lab. Different narrative contexts were presented in each activity to maintain high motivation and stimulate attention, teamwork, and collaboration among peers.

Following an adaptive paradigm, progressively more difficult activities were planned by experimenters and proposed to the classes to promote more complex competences in terms of cognitive and robot programming goals. Each week, specific cognitive and robot programming goals were proposed for the two ER-Lab sessions with Bee-Bot. Moreover, additional and optional activities with Bee-Bot were provided weekly, developed to reach the specific goals. The first 2 weeks focused on becoming familiar with the robot and improving simple visuospatial planning, the third and fourth weeks addressed complex visuospatial planning to increase working memory load through robot use, the fifth and sixth weeks focused on improving working memory abilities in response to inhibition tasks through robot use, the seventh and eighth weeks were directed at inhibiting automatic responses in set-shifting or task-switching conditions through robot use, and the ninth and tenth weeks were dedicated to using robotic programming to enhance academic skills. Details of cognitive and robot programming goals and examples of activities provided for each ER-Lab week are reported in the Supplementary Materials. Concurrently, a metacognitive approach was encouraged during ER-Lab activities, which included mentally planning complex sequences of actions before a motor act in a group context, sequential reasoning, and the ability to formulate feedback among peers. This approach promotes a problem-solving strategy based on “think before acting.” The ER-lab activities were incrementally more challenging and directed mainly toward visuospatial planning, response inhibition, working memory, and cognitive flexibility.

### Study Design

**FIGURE 2:**
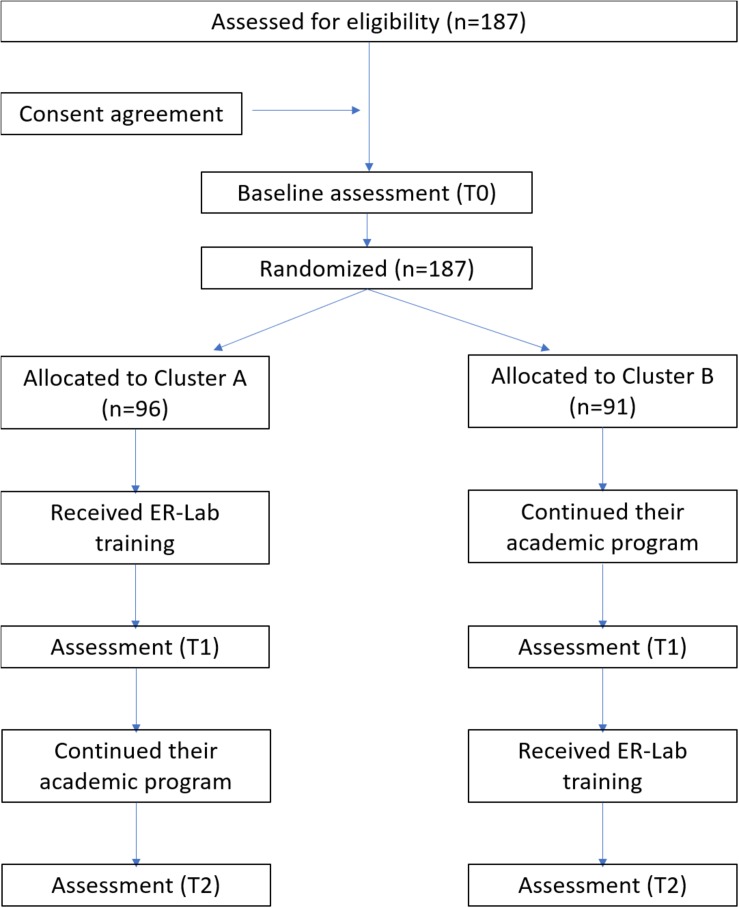
Flow diagram of the study.

According to the waitlist randomized trial design, children were randomly split into two groups (experimental condition A, *n* = 96, and experimental condition B, *n* = 91) for the sequential training rollout. Both experimental conditions were assessed by neuropsychological tests (for details, see section “Outcome Measures”) at time point T0 (September 2016). After evaluation, only children in experimental condition A started ER-Lab training immediately while those in experimental condition B continued their normal academic program. After 10 weeks, all children (experimental condition A and B) were re-tested at time point T1 (January 2017). After the T1 assessment, experimental condition B started ER-Lab training, while experimental condition A continued the normal academic program. After another 10 weeks, all children were retested at time point T2 (May 2017) (see Figure 2 for the Study Flow Diagram). The evaluators tested children at the three time points and recorded the data, and separate examiners collected and entered data in a database. The evaluators and examiners were blind to the study design and external to the research team.

### Outcome Measures

At each time point, children were assessed by standardized neuropsychological tests and qualitative measures of robotic programming skills. More than one test for each EF component of interest was selected to limit “task-impurity” that may have larger effects when only one measure is used. For visuospatial working memory, we chose Corsi Block Tapping and Matrix Path tests that require maintaining and updating information organized in a visual matrix and thus, are similar to planning robot navigation on carpets organized in a 15 × 15 matrix. While Corsi Block Tapping measures the maintenance of a global pattern in visual working memory, Matrix Path forces step-by-step information updating, thus loading working memory processes more than Corsi. Both Corsi Block Tapping and the Matrix Path test have been extensively reported in the literature and satisfy psychometric proprieties, including construct validity (Mammarella et al., 2008). Within the inhibition domain, we chose three tests, Inhibition, Little Frogs, and Pippo Says, that focus on response inhibition, rather than interference control, because the ER-Lab activities require children to inhibit automatic responses across different verbal domains (measured mainly by the Inhibition test), visual-motor domains (Little Frogs test), and motor domains (Pippo Says test). Raw scores were collected for each quantitative or qualitative measure of the administered subtests.

#### Visuospatial Memory

(1) Forward Corsi Block Tapping subtest (BVS test). This test assesses the child’s visuospatial memory amplitude (called “span”) by evaluating the longest visuospatial sequence the child can remember. The visuospatial sequence is represented by a sequence of blocks positioned on a plastic board that the examiner touches and the child has to touch in the same order. The longest sequence of blocks correctly repeated represents the obtained span and serves as the final test score. The subtest’s validity and reliability (*r* = 0.60) are reported in the BVS-Corsi manual (Mammarella et al., 2008).

#### Executive Functions

##### Visuospatial working memory

(1) Backward Corsi Block Tapping subtest (BVS test). This test is similar to the preceding test but assesses visuospatial working memory abilities by asking the child to both maintain and elaborate the visuospatial information. The child has to touch the blocks in the reverse order of the examiner’s touches, starting with the last block and ending with the first. The longest sequence of blocks correctly repeated in the reverse order represents the obtained backward span and represents the final score. The subtest’s validity and reliability (*r* = 0.74) are reported in the BVS manual (Mammarella et al., 2008).
(2) Matrix Path (BVS-Corsi). This test assesses the ability to update visuospatial information based on verbal commands held in short-term memory. The child is asked to indicate in a matrix the final destination reached following a sequence of progressively longer steps read by the examiner. The final score is the sum of correct responses. The subtest’s validity and reliability (Cronbach’s α = 0.85) are reported in the BVS-Corsi manual (Mammarella et al., 2008).

##### Prepotent response inhibition and interference control

(1) The Inhibition subtest (NEPSY-II test) has two conditions: the control (naming) condition in which the child denominates a sequence of two alternating figures and the inhibition condition, where the child denominates the two figures exchanging the label (for example, he has to say “circle” when he sees a square and vice versa). By evaluating the number of errors, self-correcting responses, and time for each condition, this test measures the ability to inhibit automatic verbal responses. The subtest’s validity and reliability (Pearson *r* coefficients ranged from 0.21 to 0.91 across all aged groups) are reported in the NEPSY-II clinical and interpretive manual (Korkman et al., 2007; Urgesi and Fabbro, 2011).
(2) In the Little Frogs subtest (BIA), the child marks steps on a small staircase drawn on a paper every time he or she hears the word “go” but must stop as soon as he or she hears the word “no-go.” The score is the number of correct responses. This test primarily evaluates visual-motor response inhibition in the context of selective and sustained attention. The subtest’s validity and reliability (percentage agreement 78%) are referred to the “Walk, don’t walk” test included in the Test of Everyday Attention for Children, as mentioned in the BIA manual (Marzocchi et al., 2010).
(3) The Pippo-Says test (a modified version of Simon-Says) is composed of two conditions: in the first, the child is instructed to do a body action only when Pippo gives the command, and thus the phrase starts with “Pippo says”; in the second condition the examiner performs all the commands in front of the child regardless of whether “Pippo says,” resulting in increased interference. The score is the number of correct responses. This test measures motor inhibition and interference control and the ability to switch between two task conditions (cognitive flexibility). The statistical characteristics and reliability (kappas > 0.90) of the test are reported by Marshall and Drew (2014).

#### ER-Lab Test

**FIGURE 3:**
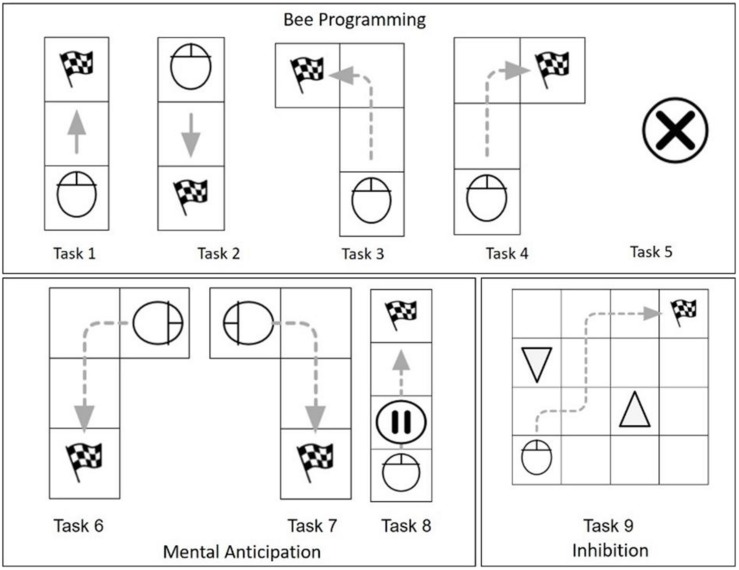
ER-Lab test.

To assess improvements in Bee-Bot programming skills, we used a test created in our previous pilot study (Di Lieto et al., 2017). The test comprises nine tasks divided into three clusters: (1) Bee programming (tasks one to five) assesses Bee-Bot use knowledge, (2) mental anticipation (tasks six to eight) assesses the ability to plan complex visuospatial pathways using Bee-Bot, and (3) inhibition (task nine) assesses the inhibition abilities elicited by Bee-Bot use (Figure 3).

Children were asked to perform the nine tasks at the beginning, after 5 weeks, and at the end of ER-Lab training. For each task, zero points were awarded if the child failed to reach the final goal, a half-point was awarded if concrete help (such as anticipating correct navigation by using their own hand or the Bee-Bot) was used to reach the goal, and one point was awarded if no concrete help was necessary.

### Statistical Analysis

Statistical analyses were performed using R, the R Project for Statistical Computing software package, version 3.6.0, with a significance level of 5%.

The effect of the training was tested by separate linear mixed-effects models for each outcome measure, with ER-Lab training and experimental condition (A or B) as fixed factors and subject ID as a random factor, in a repeated measures design. Simultaneous tests for general linear hypotheses were used to test the following two *post hoc* contrast variables for determining neuropsychological differences during ER-Lab training under both experimental conditions:

• Training effect, calculated by adding delta changes for time points T1 and T0 for experimental condition A and delta changes for time points T2 and T1 for experimental condition B
• Within-baseline effect, calculated by adding baseline delta changes in experimental condition B (T1-T0 for experimental condition B) and follow-up in experimental condition A (T2-T1 for experimental condition A).

Effect size (Cohen’s *d*) was calculated compared pre- and post-training performances in each outcome measure in both experimental conditions.

Repeated measure ANOVAs, with *post hoc* Bonferroni corrections, were performed to test differences in ER-Lab tests at the beginning, middle, and end training sessions.

A *post hoc* correlation analysis was performed between the training effect (delta changes for T1–T0 for experimental condition A and for T2–T1 for experimental condition B) in the outcome measures that showed significant improvement after the training; the delta changes in each ER-Lab test cluster (first three sessions/last three sessions) were checked by Spearman rho non-parametric tests for bivariate correlations.

## Results

Descriptive statistics for time points T0, T1, and T2 for each neuropsychological outcome are reported in Table 2.

**TABLE 2:** Mean and standard deviation on T0, T1, and T2 time points for each neuropsychological outcome in experimental conditions A and B.

Neuropsychological outcome	Experimental	T0	T1	T2
	condition	Mean ± SD	Mean ± SD	Mean ± SD
Forward Corsi Block Tapping test	A	3.03 ± 0.75	3.60 ± 0.84	3.77 ± 0.73
	B	3.08 ± 0.79	3.63 ± 0.68	3.76 ± 0.70
Backward Corsi Block Tapping test	A	2.09 ± 0.80	2.78 ± 0.88	2.96 ± 0.88
	B	2.24 ± 0.90	2.49 ± 0.79	2.95 ± 0.95
Matrix Path test	A	4.89 ± 4.20	8.42 ± 5.21	10.53 ± 5.60
	B	4.12 ± 3.60	6.57 ± 4.24	8.85 ± 4.53
Time in naming condition	A	94.01 ± 23.07	70.74 ± 13.66	63.25 ± 11.17
	B	92.98 ± 20.42	72.44 ± 15.12	64.81 ± 11.45
Errors in naming condition	A	2.00 ± 2.30	1.11 ± 2.34	0.98 ± 1.31
	B	1.44 ± 1.94	1.08 ± 1.54	0.86 ± 1.29
Self-correcting responses in naming condition	A	2.69 ± 2.12	1.20 ± 1.37	1.03 ± 1.24
	B	2.35 ± 1.95	1.49 ± 1.43	1.34 ± 1.47
Time in inhibition condition	A	126.29 ± 29.11	98.69 ± 22.22	88.49 ± 17.35
	B	130.71 ± 28.68	102.74 ± 22.15	91.54 ± 17.21
Errors in inhibition condition	A	6.75 ± 6.41	3.46 ± 4.12	2.68 ± 3.33
	B	4.70 ± 4.53	2.79 ± 3.58	1.88 ± 2.16
Self-correcting responses in inhibition condition	A	4.70 ± 2.83	3.32 ± 2.73	3.40 ± 3.10
	B	4.52 ± 2.53	3.25 ± 2.69	3.42 ± 2.47
Little Frogs test	A	9.72 ± 5.51	13.96 ± 4.53	14.38 ± 3.84
	B	9.64 ± 4.92	12.04 ± 4.95	14.65 ± 4.23
Pippo Says test	A	7.22 ± 2.06	8.39 ± 1.69	8.64 ± 1.45
	B	7.19 ± 2.10	8.27 ± 1.66	8.89 ± 1.56

*Legend: T0 represents pre-training assessment in experimental condition A and baseline assessment in experimental condition B; T1 represents post-training assessment in experimental condition A and pre-training assessment in experimental condition B; T2 represents follow-up assessment in experimental condition A and post-training assessment in experimental condition B.*

### Differences Between Experimental Conditions at Baseline

Experimental conditions A and B did not differ on chronological age (*t*(185) = 1.37, ns) or gender (χ^2^(1) = 0.12, ns). No significant differences in any neuropsychological outcome measures were found between the two experimental conditions at T0.

### Effect of ER-Lab Training on EF

As shown in Table 3, improved performance at the end of training was found in the Matrix Path test, in time, errors, and self-correcting responses in the naming and inhibition conditions, and in the Little Frogs test. As showed in Table 4, a moderate effect size was found in Matrix Path, Self-correcting responses in naming condition, Time in Inhibition condition and Little Frogs tests. A large effect was found in Time in naming condition test. No statistical differences emerged in the Forward and Backward Corsi Block Tapping and Pippo Says tests.

**TABLE 3:** Results of mixed-effects model and *post hoc* comparisons on delta changes in all children.

Neuropsychological outcome	Within-baseline effect^+^	*Post hoc* comparison	Training Effect^®^	*Post hoc* comparison
		
	Estimated mean (CI)	*p*	Estimated mean (CI)	*p*
Forward Corsi Block Tapping test	0.05(−1.03,1.13)	0.992	−1.03(−2.55,0.49)	0.211
Backward Corsi Block Tapping test	−1.29(−2.54,−0.03)	0.044*	1.61(−0.15,3.37)	0.075
Matrix Path test	0.36(−6.55,5.83)	0.985	10.29(−1.60,18.99)	0.017*
Time in naming condition	1.51(−16.78,19.81)	0.971	−180.08(−205.86,−154.30)	0.001*
Errors in naming condition	2.00(−1.02,5.03)	0.221	−6.94(−11.18,−2.71)	0.001*
Self-correcting responses in naming condition	2.07(−0.32,4.46)	0.096	−9.58(−12.94,−6.22)	0.001*
Time in inhibition condition	−16.26(−41.66,9.14)	0.245	−212.83(−248.63,−177.036)	0.001*
Errors in inhibition condition	5.42(−0.76,11.60)	0.091	−23.63(−32.29,−14.96)	0.001*
Self-correcting responses in inhibition condition	−0.57(−3.59,4.73)	0.912	−9.23(−15.06,−3.40)	0.001*
Little Frogs test	−4.91(−10.99,1.18)	0.125	8.78(0.23,17.33)	0.043*
Pippo Says test	0.07(−2.32,2.47)	0.997	−3.31(−6.68,0.06)	0.055

Legend: ^+^Within-baseline effect, differences during normal academic program in both experimental conditions, calculated by adding delta changes at baseline in experimental condition B (T1–T0 for experimental condition B) and in follow-up in experimental condition A (T2–T1 for experimental condition A); ^®^ Training Effect, differences during e-Rob training in both experimental conditions, calculated by adding delta changes for time points T1 and T0 for experimental condition A and delta changes for time points T2 and T1 for experimental condition B; * statistical significant differences (*p* < 0.05).

**TABLE 4:** Effect size values (Cohen’s *d*) in each outcome measure in both experimental conditions.

Neuropsychological outcomes	Cohen’s *d*
Forward Corsi Block Tapping test	0.46
Backward Corsi Block Tapping test	0.65
Matrix Path test	0.63
Time in naming condition	0.80
Errors in naming condition	0.28
Self-correcting responses in naming condition	0.50
Time in inhibition condition	0.77
Errors in inhibition condition	0.43
Self-correcting responses in inhibition condition	0.23
Little Frogs test	0.69
Pippo Says test	0.49

**FIGURE 4:**
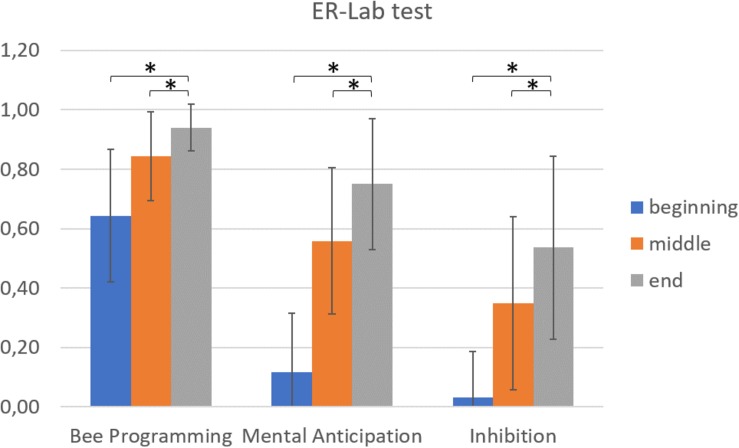
Visual representation and significant differences (^∗^*p* < 0.05) across ER-Lab test perfomances in the beginning, middle and end sessions.

For the ER-Lab tests (Figure 4), experimental condition A showed a positive learning trend in the Bee programming cluster (*F*(2,172) = 118.6, *p* < 0.001), with performances significantly higher at the end of ER-Lab training with respect to both the beginning (*t*(88) = −13.5; *p* < 0.001) and middle (*t*(87) = −6.6, *p* < 0.001) sessions. Positive trends were also found in the mental anticipation cluster (*F*(2,174) = 437.4, *p* < 0.001), with significant benefits of training evident at the end with respect to the beginning (*t*(89) = −28.3, *p* < 0.001) and middle (*t*(88) = −9.7, *p* < 0.001) sessions. As in previous clusters, inhibition cluster performances significantly improved during ER-Lab training (*F*(2,168) = 89.0, *p* < 0.001), with higher scores at the end compared to the beginning (*t*(89) = −12.4, *p* < 0.001) and middle (*t*(84) = −2.2, *p* = 0.03) sessions. Similar results were found in ER-Lab test performances in experimental condition B. A positive learning trend emerged in the Bee programming (*F*(2,168) = 139.09, *p* < 0.001), mental anticipation (*F*(2,168) = 452.34, *p* < 0.001) and inhibition (*F*(2,174) = 306.39, *p* < 0.001) clusters, with performances significantly higher at the end of ER-Lab training compared to both the beginning (*p* < 0.001) and middle (*p* < 0.001) sessions in all clusters.

*Post hoc* correlation analysis showed a negative correlation between the mental anticipation cluster and the training effect for the delta changes in self-correcting responses in the naming condition (rho = 0.15, *p* = 0.02). No other significant correlations were found.

## Discussion

The main findings of this study suggest that intensive, enjoyable, and challenging ER activities presented with incremental difficulty of cognitive and robot programming goals can improve visuospatial working memory and inhibition processes in young typically developing children.

Our results were consistent with previous qualitative studies (Benitti, 2012; Alimisis, 2013); however, this is the first study to demonstrate quantitative positive effects of ER activities using a rigorous and scientific approach. Post ER-Lab, performance in assessed ability to actively manipulate relevant information in visuospatial working memory and suppress an automatic response in favor of a goal-appropriate action improved significantly compared to the control condition.

The assessments showing significant improvement included the Matrix Path test, which measures enhanced visuospatial working memory abilities, the number of correct responses in the Little Frogs test, and improved time, errors, and self-correcting responses in the inhibition test. However, not all measures showed significant ER-Lab effects: no significant changes were found in Corsi Block Tapping or the Pippo Says test.

These differences are not easily interpretable, because they might result from several factors, such as EFs task impurity, the EFs structure model, suitability for first-grade children, and the construct validity of each measure. Nevertheless, some hypotheses may be advanced: within the working memory domain, robot programming requires active manipulation of sequential overt and covert verbal instructions and integrating them with visuospatial updates based on the robot’s position. Therefore, it is plausible that this type of exercise may result in better performance in a test such as Matrix Path that requires online integration and updating of verbal-visual information. Although the Corsi Block tests may also be solved by global visual perception strategies that mentally link the target blocks, Matrix Path seems to require step-by-step processing and may therefore be more affected by training that involves updating of the working memory. Thus, ER-Lab seems to affect the ability to construct a mental visuospatial model from verbal input and then operate on it.

Moreover, during ER-Lab activities, children had to reach a predetermined goal by planning and providing the correct commands to Bee-Bot while simultaneously respecting the rules and waiting for their turn. Therefore, ER-Lab tasks may have favored the ability to inhibit motor responses, as measured by the Little Frogs test, and control cognition and attention interference, as measured by the Inhibition test, which showed that a decreased number of self-correcting responses in a naming task was significantly related to increased ability to plan complex visuospatial pathways with Bee-Bot. It may be that the Little Frogs and Inhibition tests differ from the Pippo Says test, which showed no training effect, in that they require more child autonomy in selective and sustained attention. Consistent with this hypothesis, a ceiling effect was found in the easier condition of the Pippo Says task at the pre-training assessment.

These findings, in part, confirm the results of our previous study (Di Lieto et al., 2017, 2019), which showed improved performance in visuospatial working memory and inhibition, and are also consistent with recent literature on EF interventions in childhood showing that increasingly challenging working memory and inhibition exercises are crucial for cognitive development (Diamond and Lee, 2011; Wass et al., 2012; Wass, 2015; Spencer-Smith and Klingberg, 2016). Moreover, these two EF components are often impaired in neurodevelopmental disorders such as attention deficit with hyperactivity disorder (De La Fuente et al., 2013), specific learning disabilities (Kudo et al., 2015), autism spectrum disorders (Chen et al., 2016), and cerebral palsy (Bottcher et al., 2009). They are called “tools for learning” because they may represent early developing cross-modal basic processes that affect subsequent development of superior cognitive functions (Wass et al., 2012) and academic skill acquisition (Bull and Scerif, 2001; Blair and Razza, 2007; Van de Weijer-Bergsma et al., 2015).

This study has some limitations: first, EF tests were chosen according to the type of training used rather than specific cognitive theory; thus, the findings do not reference or link to a single theoretical framework. Moreover, the complexity of the EFs construct introduces task impurity effects that increase the difficulty of measuring separate EF components (Miyake et al., 2000). In addition, the study did not assess the distant effects of ER-Lab, such as eventual improvements in other cognitive or academic domains beyond EFs.

Given these limitations, future research is needed to confirm the results, compare ER training to other types of EF trainings, and better define and clarify its efficacy with respect to specific EF structure models.

## Conclusion

This study provides the first quantitative evidence for the positive effects of ER-Lab activities on EFs, especially working memory and inhibition, and supports using ER-Lab as an evidence-based methodology (Klingberg et al., 2005; Rueda et al., 2005; Thorell et al., 2009; Wass et al., 2011; Wass, 2015; Diamond and Ling, 2016) to improve Efs in the early school years. ER-Lab, methodologically speaking, may be halfway between telerehabilitation (Klingberg et al., 2005; Thorell et al., 2009; Grunewaldt et al., 2013) and play-based approaches (Traverso et al., 2015) as a valid tool for improving Efs during childhood. Moreover, our results suggest the importance of early intervention and the potential of carrying out this type of training in a classroom environment to directly improve school performance and assist children with EF weaknesses in an ecological, inclusive and social context.

## Data Availability Statement

The datasets generated for this study are available on request to the corresponding author.

## Ethics Statement

The studies involving human participants were reviewed and approved by the Pediatric Ethics Committee of the Tuscany Region. Written informed consent to participate in this study was provided by the participants’ legal guardian/next of kin.

## Author Contributions

MD, CP, EC, EI, and GS designed the RCT, collected the data, conducted the statistical analyses, and interpreted the results. MD and CP wrote the first draft of the manuscript. EC, EI, and GS critically revised the manuscript. FC, PD, and GC participated in designing the RCT and critical revision of the manuscript. All authors approved the final version of the manuscript.

## Conflict of Interest

The authors declare that the research was conducted in the absence of any commercial or financial relationships that could be construed as a potential conflict of interest.
