# Supplementary material for: Empowering Executive Functions in 5- and 6-Year-Old Typically Developing Children Through Educational Robotics: An RCT Study
Source: Front Psychol. 2020 Feb 5;10:3084. doi: 10.3389/fpsyg.2019.03084 (PMC7012808; doi:10.3389/fpsyg.2019.03084)
Supplement: Supplementary file 1 [file Table_1.pdf]

# *Supplementary Material*

**Details of cognitive and robot programming goals and example of activities for each ER-Lab week**

| ER-Lab | Goals | Examples of activity |
|---|---|---|
| **Week 1** | **Cognitive**:<br>Familiarization of Bee-bot use, simple visuo-spatial planning<br>**Robot programming:**<br>To reach a target placed two footsteps forward (1), or two footsteps forward and one on the right (2) or on the left (3); to understand "clear" command (4)<br>1 2 3<br>4<br>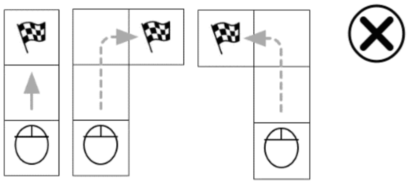 | <u>*Bee visits the city*</u><br>Bee arrives in the city! Let's make a tour of the city representing on the carpet reaching different targets (the bar or school, or restaurant). |
| **Week 2** | **Cognitive**:<br>More complex visuo-spatial planning<br>**Robot programming:**<br>To understand "pause" command<br>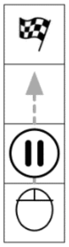 | <u>*Happy birthday, Bee!*</u><br>Bee has organized a birthday party and has to deliver the invitations to it friends. But attention, some obstacles (a car, a cat or others) pass on the streets and must be avoided, using "pause" command at the right time |
| **Week 3** | **Cognitive**:<br>Working memory and visuo-spatial planning<br>**Robot programming:**<br>To reach a target placed footsteps backwards (1), or at the end of a brief pathway concerning multiple rotations (2) | <u>*Bee is hungry!*</u><br>Bee is hungry and decides to reach some flowers to pick up pollen. The flowers are represented by geometric shapes on the carpet with different colours, shapes and sizes. The child has to follow |

| | | |
|---|---|---|
| | 1 2<br>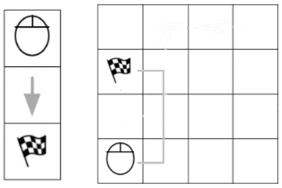 | instructions given by a teacher with an incremental challenging (for example a simple instruction is “the best pollen is in red flowers” while a hard command is “the best pollen is in yellow, big flowers and in red little flowers”. |
| **Week 4** | **Cognitive**:<br>working memory and inductive logical reasoning<br>**Robot programming:**<br>To reach a target placed at the end of a complex pathway (characterizing by much steps forward or/and backward, on the right and/or left, using “pause” command). | <u>*Bee’s dance*</u><br>Bee wants to learn a new dance. The teacher gives hidden commands to the Bee-bot and shows the final dance to the children. They have to guess the correct dance steps given. |
| **Week 5** | **Cognitive**:<br>working memory and inhibition<br>**Robot programming:**<br>To reach a target placed at the end of a complex pathway, considering Bee-Bot’s perspective taking and memory. | <u>*Finding Bee-Bot!*</u><br>Bee wants to meet a friend, but doesn't remember the road to reach him, and makes often one wrong step. The teacher gives a wrong command to Bee; thus, the child has to consider it before to program Bee-Bot without pressing “clear” command. |
| **Week 6** | **Cognitive**:<br>working memory and inhibition<br>**Robot programming:**<br>To reach one or two targets placed at the end of a complex pathway to avoid some obstacles.<br>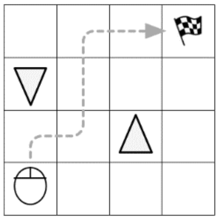 | <u>*Be careful to buds!*</u><br>Bee has to pick up more pollen as possible, according to a command given by the teacher, moving on the flowers representing as geometric shapes on the carpet. But be careful, flowers with |

| | | |
|---|---|---|
| | | triangle shape are buds and must be avoided! |
| **Week 7** | **Cognitive**:<br>Inhibition and cognitive flexibility<br>**Robot programming:**<br>To follow a high number of commands given | <u>*Bee meets Pinocchio and Jiminy Cricket*</u><br>Two new characters are presented: Pinocchio, who is lying, and Jiminy Cricket, who tells the truth. When Pinocchio gives the command, the child has to perform the opposite command (e.g. if Pinocchio says 2 steps forward, the child has to perform 2 steps backwards), while if Jiminy Cricket gives the command, the child follows it because it is correct. Toward the end of the activity, the characters' roles are inverted. |
| **Week 8** | **Cognitive**:<br>Inhibition and cognitive flexibility<br>**Robot programming:**<br>To follow a high number of commands given | <u>*Bee play by Goose game!*</u><br>A final target is posed on the carpet and children pick some notes with commands written. If the note is green, the child has to follow the command; if the note is red, child as to perform the opposite command of what written; if the note is black, the child misses a turn. |
| **Week 9** | **Cognitive**:<br>Phonological working-memory, alpha-numeric ability<br>**Robot programming:**<br>To follow a high number of commands given or to reach one or two targets placed at the end of a complex pathway | <u>*Bee learns to write!*</u><br>Every child writes his/her own name with Bee-bot reaching the corresponding letters on the carpet and pressing "pause" command when arrives on them. |
| | **Cognitive**: | <u>*Bee learns to calculate!*</u> |

| | | |
|---|---|---|
| **Week 10** | Working memory and numeric ability<br>**Robot programming:**<br>To follow a high number of commands given or to reach one or two targets placed at the end of a complex pathway | Children have to perform some arithmetic calculation, reaching first the numbers of the calculation and then the result with Bee-bot on the carpet. |
